# Preservice Special Education Teachers’ Acceptance of an AI-Based Microteaching Evaluation and Feedback System: A Pre–Post UTAUT Mixed-Methods Study

**DOI:** 10.3390/bs16101818

**Published:** 2026-10-04

**Authors:** Gayoung Lee, Youngjin Yoo, Seolhee Lee

**Affiliations:** 1 Department of Special Education, Baekseok University, Cheonan 31065, Republic of Korea; gayounglee@bu.ac.kr; 2 Office of Educational Innovation, Pai Chai University, Daejeon 35345, Republic of Korea; treplok@pcu.ac.kr

**Keywords:** artificial intelligence, microteaching, preservice special education teachers, instructional evaluation, UTAUT, expectation confirmation, mixed methods

## Abstract

We examined preservice special education teachers’ acceptance of an AI system that supports the evaluation of, and feedback on, their own teaching demonstrations. The system analyzes recorded demonstrations against a structured rubric with time-stamped behavioral evidence, allows users to review and revise the AI evaluation through chatbot dialogue, and then generates personalized feedback. We used a convergent mixed-methods design informed by the Unified Theory of Acceptance and Use of Technology (UTAUT) and interpreted through an expectation-confirmation lens. Undergraduate students at a private university in South Korea completed a 23-item UTAUT-based questionnaire before and after a semester of use of the system in two required courses (valid pre-use responses *N* = 42; posttest *N* = 65; matched pairs *n* = 33), and 20 students were interviewed across the semester’s two demonstration cycles. Acceptance was uniformly high before use (composite means 4.22–4.63 on a 5-point scale) and did not decline after a semester of use; post hoc, exploratory one-sided non-inferiority tests rejected a decline of *d* = −0.35 or larger for every factor (all *p* ≤ 0.007). Performance expectancy increased modestly (*d* = 0.36; *t*-test *p* = 0.047, Wilcoxon *p* = 0.055, Holm-adjusted *p* = 0.282), a change that is best read as suggestive rather than established. At posttest, a regression of intention on the four UTAUT factors accounted for 62% of the variance, but effort expectancy (β = 0.37) showed the most consistent evidence of association with intention across classical, heteroscedasticity-robust, bootstrap, and influence-adjusted specifications; the association for social influence (β = 0.39 under classical estimation) was not robust to the estimator, performance expectancy was significant only when one influential case was excluded, and facilitating conditions were nonsignificant throughout. Facilitating conditions were, as the original model specifies, strongly associated with use behavior (β = 0.65) rather than with intention. In interviews, participants described their sustained acceptance in terms of evidence-grounded, time-stamped feedback, score discrepancies that prompted questioning and reflection, uptake into lesson planning and re-teaching, and the system’s role as an accessible complement to scarce instructor feedback, and they located the limits of that acceptance in context-recognition errors, feedback inconsistency, and interface friction. Taken together, the findings suggest that high pre-use expectations of AI-supported evaluation need not erode over a semester of sustained use when the system continuously displays the grounds of its judgments, and that ease of use, rather than perceived usefulness, is the factor showing the most consistent evidence of association with continued-use intention.

## 1. Introduction

Microteaching has long been established as a core pedagogical method in teacher preparation programs worldwide. Preservice teachers rehearse instructional decision-making by delivering scaled-down lessons under conditions that reduce and buffer the demands of a real classroom (Allen & Ryan, 1969; Grossman et al., 2009). The educational value of microteaching, however, depends decisively on the quality of the evaluation and feedback provided after each demonstration. Under typical operating conditions, a single instructor must observe, score, and comment on dozens of demonstrations within compressed time frames, and the predictable consequences are rater fatigue, evaluations that drift with individual emphases, feedback that arrives long after the demonstration, and comments too brief to guide concrete revision (Hattie & Timperley, 2007).

These constraints are amplified in special education. Preservice special education teachers must demonstrate not only general teaching skills but also competencies specific to instructing students with disabilities, including applying universal design for learning, using evidence-based practices, interacting with and individualizing for diverse learners, collaborating with related professionals, intervening in challenging behavior, and using current instructional media (S. H. Lee et al., 2024). Each of these requires evaluation against multidimensional criteria that are cognitively demanding for a human rater to apply consistently (Bejar, 2012). When feedback on these competencies is delayed or superficial, opportunities to correct misconceptions about teaching students with disabilities are lost at precisely the stage of professional development when they are most correctable.

Three features of this situation—the delay between demonstration and feedback, the inconsistent application of multidimensional criteria across a large cohort, and the limited volume of comment any single rater can produce—define the problem that computational support might address. Advances in multimodal large language models (LLMs) now make a credible response to each of them possible: such models can process video, audio, and text in an integrated fashion, link observed behaviors to rubric criteria with time-stamped evidence, and return criterion-referenced comments within minutes rather than weeks (Celik et al., 2022; G. Lee et al., 2025; U. Lee et al., 2024; Zawacki-Richter et al., 2019).

Systems built on these capabilities need not position AI as a replacement judge, and the system examined in this study was deliberately designed around human–AI collaboration: the AI produces an evidence-grounded first-pass evaluation, and the user then reviews, questions, and adjusts that evaluation through chatbot dialogue before personalized feedback is generated, so that the final judgment integrates computational analysis with human contextual interpretation (Holstein et al., 2019; G. Lee et al., 2025). The design is also grounded in feedback-literacy research, which holds that feedback should present strengths and weaknesses in balance and be delivered in a form that learners can take up and act on (Carless & Boud, 2018). The system itself is described in Section 3.3; what concerns us here is that its educational potential is only realized if its intended users come to accept it, and if that acceptance survives the friction of repeated, consequential use. Acceptance is not the same as use, and neither guarantees productive use (Straub, 2009); but two decades of technology acceptance research consistently show that adoption depends on beliefs about a system’s usefulness and ease of use, the social environment surrounding its use, and the available support infrastructure (Davis, 1989; Scherer et al., 2019; Venkatesh et al., 2003).

The stakes of this acceptance extend beyond the tool itself. Teacher digital competence is now understood not as a set of technical skills but as a professional capability exercised within a pedagogical and ethical context, encompassing the judgment to use digital resources appropriately and to recognize their limits (Falloon, 2020). An AI system that evaluates a preservice teacher’s own performance sits squarely in that territory: using it well requires interpreting an algorithmic judgment, weighing it against one’s own reading of the lesson, and deciding what to accept. Preservice teachers who form habits of uncritical acceptance at this stage are likely to carry them into settings where AI-generated evaluation of pupils, rather than of themselves, is at issue. Teacher preparation is also the opening phase of a professional career in which evaluative tools will continue to change, so what is learned here is less a particular system than a disposition toward evaluative technology that must be sustained through continuing professional learning. This is why acceptance is worth studying in its own right, and why acceptance that is uncritical would not be a good outcome.

Acceptance research faces a specific difficulty at the present moment. As public discourse on generative AI has proliferated, preservice teachers often arrive at their first structured encounter with an educational AI tool already holding elevated expectations, and single-time-point acceptance studies cannot tell whether such expectations survive extended experience with a system used for consequential coursework. Expectation-disconfirmation theory would predict that inflated expectations collapse on contact with an imperfect system (Bhattacherjee, 2001; Oliver, 1980). Whether initial acceptance is maintained, strengthened, or eroded through actual use carries direct implications for how teacher educators integrate AI into their curricula, and change of this kind can only be established with a pre–post design. Longitudinal designs of this kind remain rare in research on the acceptance of educational AI (Granić & Marangunić, 2019).

This study addresses that gap in a context where AI-based evaluation carries high educational stakes: a special education teacher preparation program at a Korean university in which an AI system was used across a full semester for analyzing teaching demonstrations, rubric-based evaluation, and personalized feedback. Throughout this article we describe that use as consequential rather than optional: the teaching demonstrations the system evaluated were themselves graded coursework, although the system’s own output did not contribute to students’ grades (Section 3.6). It does so as the direct continuation of the system’s development program: the development study, based on an expert-only exploratory validation, explicitly identified large-scale empirical research with actual user groups as its necessary next step (G. Lee et al., 2025). Grounded in the Unified Theory of Acceptance and Use of Technology (UTAUT) (Venkatesh et al., 2003) and interpreted through an expectation-confirmation lens (Bhattacherjee, 2001), we posed the following research questions:

RQ1. What is the level of preservice special education teachers’ acceptance of the AI-based microteaching evaluation and feedback system before and after a semester (15 weeks) of sustained use in required coursework, and how does it change?

RQ2. After a semester of such use, which UTAUT factors (performance expectancy, effort expectancy, social influence, facilitating conditions) are associated with continued-use intention?

RQ3. How do preservice special education teachers describe their acceptance of the system and its limits after sustained use?

## 2. Theoretical Background

### 2.1. Microteaching and Its Challenges in Teacher Preparation

Since its development at Stanford in the 1960s, microteaching has been adopted internationally as a core method of teacher preparation (Allen & Ryan, 1969). Its rationale is that decomposing teaching into practicable performances and coupling them with immediate feedback accelerates the acquisition of teaching skills. Meta-analyses confirm feedback as one of the most powerful influences on learning, but only when it is specific, timely, and actionable (Hattie & Timperley, 2007).

In practice, the feedback component of microteaching is the most vulnerable part of the method. Performance ratings are systematically vulnerable to halo effects, in which an overall impression contaminates judgments of distinct criteria, and to rater drift, in which a rater’s application of the scale shifts across a long sequence of ratings; both are well documented in the performance-assessment literature and are amplified when raters score many performances under time pressure (Bejar, 2012; Myford & Wolfe, 2003). The interval between demonstration and feedback routinely exceeds the window in which feedback is most useful (Hattie & Timperley, 2007). Peer feedback, the common supplement, adds perspectives but does not guarantee accuracy: studies of peer assessment in teacher education report that novice raters attend to surface features of a performance and are less consistent than experts in identifying the qualitative dimensions of teaching, so peer judgments vary in their alignment with expert judgment (Panadero & Brown, 2017).

In special education teacher preparation, the evaluation criteria are broader and more differentiated. Demonstrations must be assessed not only on general dimensions such as lesson structure and questioning, but also on individualization, accommodation of diverse learner characteristics, use of assistive technology and instructional media, and intervention in challenging behavior; a validated teaching-competency framework for preservice special education teachers spans universal design for learning, effective instruction, learner interaction, collaboration, behavioral intervention, and instructional media use (S. H. Lee et al., 2024). Research on rating complexity indicates that as the number of distinct criteria a rater must hold in mind increases, inter-rater agreement declines and raters increasingly collapse distinct criteria into a single global impression (Bejar, 2012; Myford & Wolfe, 2003). This strengthens the case for computational support in multidimensional evaluation, although it does not by itself establish that computational support delivers more accurate judgments—a question the present study does not address.

### 2.2. AI Systems for Microteaching Evaluation and Feedback

Research on artificial intelligence in education has expanded rapidly across automated assessment, adaptive tutoring, learner profiling, and related areas (Zawacki-Richter et al., 2019). In teacher education specifically, AI has been applied less to replace instruction than to support teachers’ professional tasks of planning, evaluation, and reflection (Celik et al., 2022; Salas-Pilco et al., 2022). Generative-AI-based feedback has shown promise for improving preservice teachers’ instructional design competencies (Celik et al., 2022), and multimodal LLMs extend this potential further. Because they can process video, audio, and text in an integrated fashion, they can analyze the verbal and nonverbal elements of a recorded teaching demonstration—speech, gesture, gaze, and use of instructional materials—and link observed behaviors to rubric criteria with time-stamped evidence. For example, U. Lee et al. (2024) presented a GPT-4 Vision-based classroom observation assessment, and comparable systems targeting teacher preparation contexts are beginning to emerge.

Two design commitments drawn from this literature bear most directly on the present study. Human–AI collaboration positions AI not as an authoritative automatic judge but as a partner that complements human judgment; the human evaluator interprets, questions, and revises the AI’s output on the basis of contextual and professional knowledge (Holstein et al., 2019). Such collaboration involves, as core competencies, collaborative task regulation and calibrated trust rather than blind trust (Y. J. Yoo et al., 2024), and interactive review—asking the system why a given criterion received a given score and receiving its grounds—is a representative mechanism for realizing it. The second commitment concerns feedback. Feedback-literacy research, developed primarily with higher education learners, focuses on presenting evaluation results in a form learners can readily understand and act on: effective feedback presents strengths and weaknesses in balance and includes concrete feedforward suggestions (Carless & Boud, 2018). We apply this framework to preservice teachers in their capacity as higher education learners receiving feedback on their own performance; we do not extend it to feedback directed at students with disabilities, whose communicative and cognitive profiles vary so widely that the transferability of feedback-literacy constructs to that population remains an open empirical question and lies outside the scope of this study.

Work on the cognitive risks of AI-supported learning specifies what this collaboration requires in practice, and four conditions are relevant here (Y. Yoo, 2026). First, an AI evaluation can compress the process it is meant to support: because the system supplies a judgment immediately, learners may skip the interpretation of their own performance evidence that self-assessment consists of, so the sequence must place the learner’s own judgment before exposure to the system’s. Second, generative systems produce fluent and plausible explanations that may nonetheless misread the intent or context of a lesson, so their output must remain checkable against specific evidence rather than accepted on the strength of its fluency. Third, the roles of learner, instructor, and system need to be distinguished deliberately, since a system that scores, explains criteria, and proposes improvements can silently displace the instructor’s role rather than extend it. Fourth, the outputs of such a system are interpretations rather than facts, and the recordings, transcripts, and evaluation logs they rest on raise questions of privacy and data handling that belong to the design rather than to its periphery.

Alongside these two commitments, special education pedagogical alignment supplies the content foundation that situates collaboration and feedback within special education teacher preparation: observable indicators for the application of universal design for learning, the use of augmentative and alternative communication, and the responsiveness of instruction to students’ physical, cognitive, and communicative characteristics are embedded in the evaluation rubric (G. Lee et al., 2025). This is a distinguishing feature relative to general-education AI evaluation tools.

Prior research on comparable technologies in teacher preparation underscores the importance of user acceptance. Studies of mixed reality simulation platforms, for instance, found that preservice teachers’ willingness to engage with technology-based practice environments depended heavily on perceived usefulness and usability, consistent with technology acceptance findings across educational technology (Scherer et al., 2019). However, almost no research has examined—through sustained authentic use rather than one-off exposure—acceptance of an AI system that evaluates preservice teachers’ own teaching demonstrations, a system that simultaneously implicates their self-concept as future teachers and their trust in algorithmic judgment.

### 2.3. Self-Assessment as a Competence in Microteaching

The system examined here was designed not merely to deliver judgments but to support students’ own evaluative capability, and the construct it targets deserves specification. Self-assessment in this tradition is treated as a competence rather than an act: it comprises understanding and applying evaluation criteria, searching for evidence of one’s own performance, forming a judgment anchored in that evidence, examining and integrating external information, generating internal feedback, and adjusting through reflection (Y. Yoo, 2026). So specified, self-assessment is not an opinion a student holds about a lesson but a sequence of evidence-handling operations that can be scaffolded.

This specification explains why complex performances are difficult to self-assess. A teaching demonstration unfolds in real time across speech, gesture, materials, and learner response, and the performer is simultaneously the actor and the observer, so the evidence available for self-judgment is partial and reconstructed from memory. Where evidence is scarce, judgment drifts toward global impression, and the same rater effects documented in external assessment (Bejar, 2012; Myford & Wolfe, 2003) operate on the self. Recording the demonstration and returning time-stamped behavioral evidence against explicit criteria is therefore not only a convenience of automation; it restores to the performer the evidence base that self-assessment requires. The system’s evidence-grounded output and its dialogue-based review are addressed to this problem (G. Lee et al., 2025; Y. Yoo, 2026).

We note the boundary of what the present study establishes about this construct. We measured acceptance, not self-assessment competence, and the prior study that examined agreement between students’ self-assessments and instructor assessments involved a subset of this cohort under a separate design (Section 5.2). Claims about evaluative capability in this article therefore rest on participants’ own accounts and on that prior work, not on measurement conducted here.

### 2.4. UTAUT and the Expectation-Confirmation Perspective

UTAUT integrates eight predecessor theories into four core determinants of technology acceptance. Performance expectancy (PE) is the belief that the technology will improve task performance; effort expectancy (EE) is the degree of effort required to use it. Social influence (SI) is the perceived endorsement of use by important others, and facilitating conditions (FC) refer to perceptions of the organizational and technical environment supporting use (Venkatesh et al., 2003). In the original model, PE, EE, and SI predict behavioral intention (BI), whereas FC directly predicts use behavior (UB) rather than intention. UTAUT and its extensions have been applied extensively in education, and meta-analyses confirm usefulness and ease of use as core determinants of technology acceptance among teachers and preservice teachers (Granić & Marangunić, 2019; Scherer et al., 2019).

Two specifications of our use of UTAUT should be stated at the outset. First, we employ an adapted version of the model. The four core determinants, behavioral intention, and use behavior were measured, but the original moderators—gender, age, experience, and voluntariness of use—were not, both because the sample was demographically homogeneous and because use was a course requirement rather than a voluntary choice, which removes variance on voluntariness by design. The model tested here is therefore the unmoderated core of UTAUT, and our results speak to that reduced specification only.

Second, we entered facilitating conditions as a predictor of behavioral intention even though the original model specifies FC as a determinant of use behavior rather than intention (Venkatesh et al., 2003). We did so for two reasons. Where use is institutionally mandated, students cannot decline to use the system in the present semester, so intention to continue using it beyond the course is the behavioral construct at issue, and perceptions of available support plausibly bear on that judgment—and several UTAUT extensions report FC-BI paths in educational settings (Granić & Marangunić, 2019). Because this departs from the original specification, we treat the FC-BI path as exploratory, and we additionally test the model-consistent FC-UB relationship directly (Section 4.2).

Most educational applications of UTAUT have measured acceptance at a single time point, typically after brief exposure. Such designs cannot distinguish durable acceptance from novelty effects, nor can they account for post-adoption changes in expectations. According to expectation-confirmation theory, continuance intention is determined by the extent to which expectations formed before use are confirmed by actual experience; expectations that outrun reality can produce disconfirmation, dissatisfaction, and discontinuance (Bhattacherjee, 2001; Oliver, 1980). At a moment of intense public interest in generative AI, preservice teachers’ pre-use expectations may well be elevated enough to invite disconfirmation.

We therefore measured UTAUT factors before and after a semester of sustained use in order to observe whether acceptance was maintained. It is important to be precise about the status of expectation-confirmation theory in this design. We did not measure confirmation, satisfaction, or the perceived discrepancy between prior expectations and experienced performance as distinct latent variables, and we therefore do not test the expectation-confirmation model. We use it as an interpretive lens: it specifies what a disconfirmation pattern would look like in pre–post acceptance scores, and it supplies the vocabulary in which participants’ own accounts of their experience can be read. Claims about confirmation in this article are accordingly interpretive claims resting on the interview data, not quantitative findings.

## 3. Materials and Methods

### 3.1. Research Design

This study employed a convergent mixed-methods design combining a single-group pre–post survey with semi-structured interviews. UTAUT-based acceptance was measured near the beginning of the semester, before students’ substantive use of the system (valid pre-use window: 30 September–28 October 2025), and at the end of the semester, after sustained use (16–25 December 2025). Interviews were conducted at two points corresponding to the courses’ two demonstration cycles: 10 students in mid-November 2025 after the first cycle, and 10 further students, none of whom had been interviewed in the first cycle, in mid-December 2025 after the second cycle. Because the system was integrated into required courses for the full cohort, random assignment was not possible and a single-group design was adopted; the consequences of this choice for inference are addressed in Section 6.

### 3.2. Participants and Research Context

Participants were preservice teachers enrolled in the Department of Special Education at a private university in the Chungnam region of South Korea. All were taking one of two required courses in the fall semester of 2025 in which a teaching demonstration is a central graded task (Section 3.6). The two courses enrolled 75 students in total, which is the denominator for the response rates reported below.

Participant flow was as follows. The pretest survey received 83 submissions. Two exclusions were applied. First, 37 submissions were made after the respondent’s cohort had already begun using the system; because these responses could not function as pre-use measures, they were excluded. Second, duplicate submissions were resolved by retaining each student’s earliest response. These steps yielded a valid pre-use sample of *N* = 42 (56% of those enrolled). The posttest survey yielded 66 submissions; after resolving the single duplicate, the posttest sample comprised *N* = 65 (87%). The 33 students who provided usable responses at both time points, matched by student identification number, constituted the paired sample (*n* = 33, 44% of those enrolled and 79% of the valid pre-use sample). All posttest-only analyses (RQ2) used the full posttest sample (*N* = 65).

Two features of this flow warrant caution. The matched sample is small relative to the cohort, and students who completed both surveys may differ systematically from those who completed only one—plausibly in engagement with the course and with the system—so the paired analyses may overrepresent more engaged students. The exclusion of 37 pretest responses was necessary to preserve the meaning of the pre-use measure but reduced both the pretest and the matched sample and therefore the statistical power available for RQ1 (Section 3.7 and Section 6). A sensitivity analysis using a stricter pre-use cutoff is reported in Section 3.7.

The study was approved by the Institutional Review Board of Seoul National University (IRB No. 2511/002-012), the institution at which the second author was a doctoral student and who served as principal investigator at the time the study was designed and conducted. The approval process comprised an initial submission on 15 September 2025, conditional approvals on 30 September and 20 October 2025, and final approval on 27 October 2025. Data collection for the pre-use survey began after the Board had issued its first conditional approval on 30 September 2025; final approval (No. 2511/002-012) was granted on 27 October 2025. The conditions concerned revision of the submitted protocol documents and their resubmission for re-review. The pre-use survey was therefore administered before the data-collection period specified in the approved protocol. The research team is aware of this discrepancy. Informed consent for participation and for the research use of the data was obtained from participants at the outset of data collection. Following final approval on 27 October 2025, consent was obtained again, from all participants whose data are reported here, using the consent form approved by the Board, so that the research use of data collected before that date is covered by consent on the approved form. The Board was informed of this re-consent procedure. The signed documentation for both stages is retained and available to the editorial office on request. Participation was voluntary, responses were de-identified and used only for research purposes, and a small incentive (a mobile gift voucher) was provided.

For the qualitative strand, 20 students were interviewed, 10 after the first demonstration cycle and 10 after the second. Participation in the interviews was voluntary and self-selected: all students in the two courses were invited, and those who expressed willingness were interviewed. No purposive or quota criteria were applied, and no student was approached individually. In Cycle 1 the volunteers happened to divide evenly between the two courses, five from each; in Cycle 2 the ten interviewees were students who had not been interviewed in Cycle 1. Because interviewees selected themselves, they may hold more favorable views of the system, or simply be more engaged with the courses, than the cohort as a whole; the qualitative findings should be read as the accounts of students willing to discuss the system rather than as representative of all users (Section 6).

Gender and age were not directly collected. Based on the admission-year prefix of student identification numbers, respondents in both samples belonged predominantly to the 2023 and 2022 admission cohorts, that is, third- and fourth-year students in the study semester (pretest: 17 and 16 of 41 identifiable IDs; posttest: 24 and 21 of 55), with the remainder from the 2020, 2021, and 2024 cohorts. Assuming typical university entry, estimated ages ranged from 20 to 24 years (*M* ≈ 21.7). Prior experience with AI tools was not measured, which limits our ability to characterize the sample’s baseline disposition toward AI (Section 6). The system’s development and expert usability evaluation are reported separately (G. Lee et al., 2025); the present study covers its first semester of field implementation.

### 3.3. The AI-Based Microteaching Evaluation and Feedback System

The system examined in this study is a web-based AI application supporting the evaluation of, and feedback on, preservice special education teachers’ teaching demonstrations (G. Lee et al., 2025). It was developed through design and development research (Richey & Klein, 2005), and its design principles were systematized into five components comprising seven principles and thirteen guidelines: interface design; multimodal data analysis and evaluation; human–AI collaboration; feedback; and special education pedagogical alignment (G. Lee et al., 2025). The application is implemented in Streamlit with Google Gemini 2.5 Pro as its multimodal analysis engine, supporting integrated processing of video, audio, and text, and was refined through a heuristic usability evaluation (Nielsen, 1993) by five doctoral-level experts in special education and educational technology. Across 14 usability dimensions (30 items), item means ranged from 3.4 to 5.0 with an overall mean of 4.5 on a 5-point scale, and the experts’ qualitative comments informed pre-implementation improvements including step-by-step progress guidance, display of estimated processing time, a structured score-evidence-suggestion output format, and error-recovery instructions (G. Lee et al., 2025).

**Figure 1:**
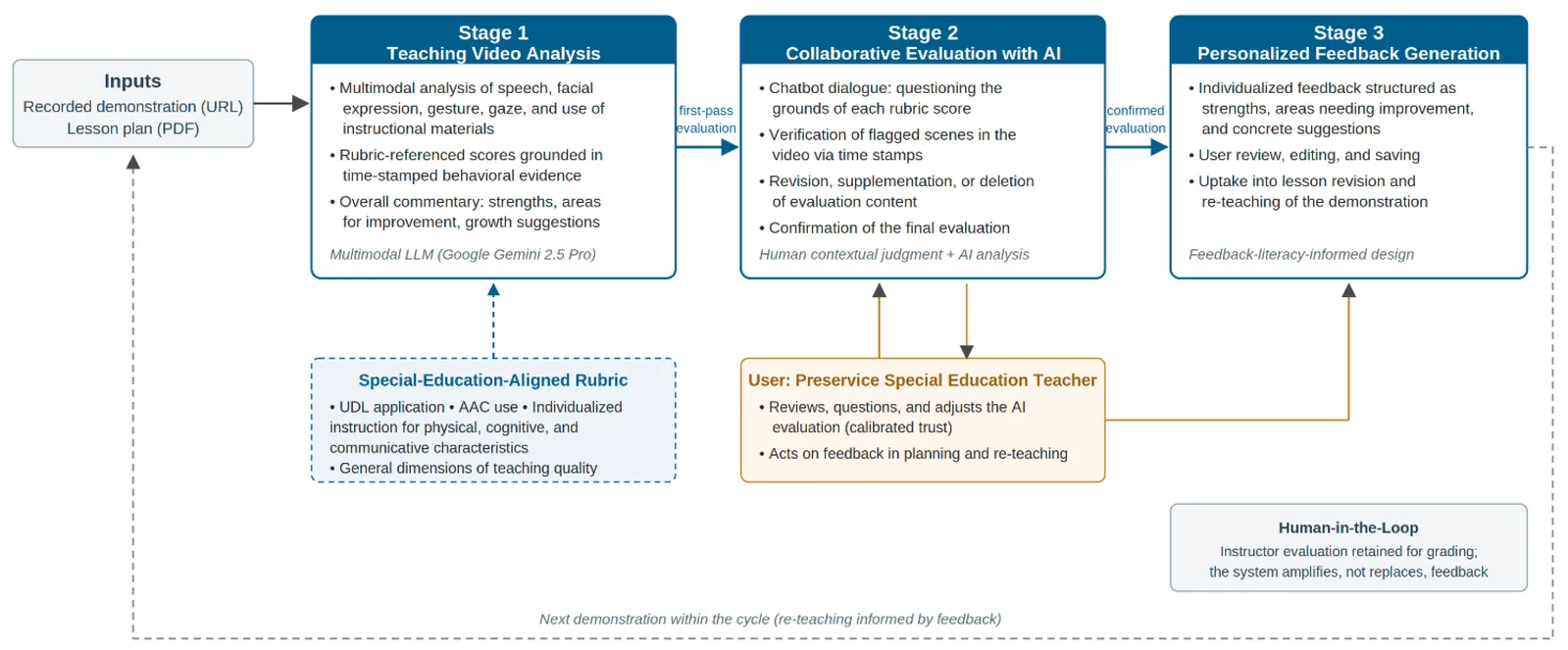
Three-stage workflow of the AI-based microteaching evaluation and feedback system. UDL = universal design for learning; AAC = augmentative and alternative communication. Solid arrows indicate the flow of inputs and evaluation outputs between stages and the user’s interactions with the system; dashed arrows indicate the rubric informing the Stage 1 analysis and the return to the next demonstration within a cycle. Redrawn in English based on the system reported in (G. Lee et al., 2025).

The system operates in three stages. First, in the video analysis stage, the user submits a link to the recorded demonstration together with the lesson plan; the system analyzes verbal and nonverbal dimensions of the performance-speech, facial expression, gesture, gaze, and use of instructional materials—and produces rubric-referenced scores in which each judgment is grounded in specific, time-stamped teacher behaviors and utterances extracted from the video, along with an overall commentary covering strengths, areas for improvement, and suggestions for professional growth. Second, in the collaborative evaluation stage, the user reviews this evidence-grounded first-pass evaluation in dialogue with a chatbot: asking why a given criterion received a given score and receiving its grounds, checking flagged moments directly in the video via time stamps, and revising, supplementing, or deleting evaluation content, so that the final evaluation layers human contextual interpretation over computational analysis. Third, in the personalized feedback stage, the system generates individualized feedback structured into strengths, areas needing improvement, and concrete suggestions on the basis of the confirmed evaluation, which the user can review, edit, and save. The evaluation rubric reflects teaching competencies specific to special education—supporting the communication of students with limited spoken language, intervening in challenging behavior, and individualizing instruction for physical and cognitive characteristics—alongside general dimensions of teaching quality (G. Lee et al., 2025). Figure 1 presents the three-stage workflow. The accuracy and validity of the scores the system produces were not evaluated in this study; our object is users’ acceptance of the system, not the quality of its judgments.

### 3.4. Instruments

Acceptance was measured with a 23-item questionnaire adapting the UTAUT instrument (Venkatesh et al., 2003) to the context of AI-based teaching demonstration evaluation. Items covered six factors: performance expectancy (PE; 6 items, e.g., “This AI tool helps me evaluate teaching demonstrations more effectively”), effort expectancy (EE; 5 items, e.g., “Learning how to use this AI tool is easy”), behavioral intention (BI; 4 items, e.g., “I intend to continue using this AI tool”), social influence (SI; 3 items), facilitating conditions (FC; 3 items), and use behavior (UB; 2 items), all rated on a 5-point Likert scale (1 = strongly disagree, 5 = strongly agree). The research team translated the original items into Korean and adapted their wording to the context of AI-based teaching demonstration evaluation; researchers with expertise in special education and educational technology then reviewed the items for content relevance and clarity of phrasing, and the items were revised accordingly before administration. Internal consistency was good at both time points (Cronbach’s α: 0.82–0.95 at pretest, 0.82–0.92 at posttest; Table 1).

Two measurement issues qualify these instruments. First, factorial validity was not established in this sample. The posttest sample (*N* = 65) is far below the case-to-parameter ratios conventionally required for confirmatory factor analysis of a six-factor, 23-item instrument, and no factor analysis was therefore attempted. Evidence for the factor structure rests on the internal consistency reported in Table 1, on the adopted structure of the source instrument (Venkatesh et al., 2003), and on the expert content review described above; it does not rest on structural evidence obtained in this sample.

Second, pre–post comparability is imperfect by construction. Following convention in expectation-confirmation research (Bhattacherjee, 2001), pretest items were phrased prospectively (“will help”) and posttest items in present or retrospective terms. This preserves the meaning of a pre-use measure but means that the two administrations are not strictly parallel forms, and measurement invariance across time could not be tested at this sample size. The use behavior factor is affected most acutely: at pretest, students had not yet used the system, so the two UB items necessarily measured anticipated rather than actual use. Pre–post comparisons on UB should therefore be read as a comparison between anticipated and self-reported enacted use, not as change in a single construct, and we do not interpret the UB change substantively.

### 3.5. Interviews

The semi-structured interviews followed a common protocol covering ten areas: recall of the overall experience of using the system; perceptions of the AI’s analysis and evaluation; experiences of evaluation accuracy and of discrepancies with one’s own judgment; score adjustment; trust in the AI evaluation; use of the criterion-specific dialogue and reflection writing; perceived changes in one’s own evaluative competency; comparison with instructor feedback; suggestions for improvement; and intention to recommend the system and preferred modes of use. Cycle 2 interviews added questions reflecting the system changes implemented between cycles, including the lesson-plan upload function. Interviews were conducted individually by the second author and audio-recorded, lasting approximately 30–60 min each, with follow-up probes used flexibly according to participants’ responses.

The second author’s position relative to participants should be made explicit, because it bears on what students were willing to say. The second author was not an instructor of either course and held no role in determining participants’ grades; he is affiliated with an institution other than the one at which the data were collected. Participants were informed that interview content would not be shared with course instructors in identifiable form and would not affect their grades. Because the interviewer was a member of the team that developed the system, students may nonetheless have moderated their criticism; the frankness of the critical accounts reported in Section 4.3 is relevant but not decisive evidence on this point (Section 6).

Interview data were anonymized and processed into theme-organized records combining summarized content with partial verbatim transcription rather than full verbatim transcripts. Quoted excerpts below are drawn from the verbatim portions of these records and are lightly edited for readability; the implications of this procedure for analytic transparency are discussed in Section 6. Interviewees are identified by cycle and number (e.g., C1-S3 is the third interviewee of Cycle 1).

### 3.6. Procedures

During the study semester, the system was integrated into two required courses taught by different instructors, both of which used the teaching demonstration as a central graded task; demonstrations addressed middle school Korean language lessons in one course and elementary integrated-subjects lessons in the other. Use was organized into a ten-week sequence within the semester: an orientation week introducing system use and data-collection procedures, followed by two four-week demonstration cycles, with the system improved in response to the first cycle introduced between cycles. Students formed small groups of four to five for scheduling purposes, but lesson planning, demonstrations, and system-supported evaluation and reflection were all completed individually. In each cycle, students wrote an individual lesson plan on a topic corresponding to their course and delivered a first recorded demonstration of approximately 10–20 min in a classroom setting; they then uploaded the demonstration video in a designated computer laboratory and conducted an AI-supported evaluation session. Drawing on the resulting reflection and improvement plan, students prepared and delivered a second recorded demonstration, followed by a second system session completed individually outside of class time. Each student thus completed up to four demonstrations and four system sessions across the semester.

Three features of this arrangement condition the interpretation of our results. Instructor evaluation of demonstrations was retained separately for grading, and the system’s output did not contribute to grades; the system supported students’ self-evaluation and reflection and expanded feedback opportunities within a human-in-the-loop structure. The demonstrations it evaluated were nonetheless graded work, so students engaged with the system under conditions of genuine consequence rather than as a low-stakes trial. Use was, however, compulsory: students could not decline to use the system, which means that reported intention cannot be read as free choice and that perceived social endorsement (SI) was measured in a setting where institutional endorsement was already unambiguous (Section 5.2 and Section 6). Finally, the two courses differed in instructor, subject matter, and the group of students enrolled, and the version of the system used in Cycle 2 differed from that used in Cycle 1. Sample sizes do not support testing course as a moderator, and pre–post responses cannot be attributed to a single stable version of the system; posttest scores should be understood as acceptance of the system as experienced across both cycles.

### 3.7. Data Analysis

For RQ1, composite scores (item means) were computed for each factor, and pre–post differences in the paired sample (*n* = 33) were tested with paired-samples *t* tests. Because score distributions were negatively skewed with pronounced ceiling effects, Wilcoxon signed-rank tests were run in parallel as a nonparametric robustness check, and both are reported. Effect sizes are Cohen’s *d* for paired data, and 95% confidence intervals are reported for all mean differences.

Three additions address the inferential problems raised by six factor-level comparisons in a small, ceiling-bounded sample. First, because RQ1 involves six comparisons, Holm–Bonferroni adjusted *p* values are reported alongside uncorrected values, and interpretation rests primarily on effect sizes and on the consistency of the pattern across factors. Second, because a nonsignificant difference does not demonstrate stability, we conducted one-sided non-inferiority tests for each factor against a lower equivalence bound of *d* = −0.35, a value chosen to correspond to a small decline that would be practically meaningful for a program deciding whether to continue an implementation. Rejecting this bound licenses the claim that acceptance did not decline appreciably; it does not license a claim of exact equivalence. The non-inferiority bound was not prespecified; these tests are therefore post hoc and exploratory, and we interpret them accordingly. Third, a sensitivity analysis repeated the RQ1 analyses restricting the pretest to responses submitted by 15 October (*n* = 28 pairs).

For RQ2, posttest BI was regressed (OLS) on posttest PE, EE, SI, and FC in the full posttest sample (*N* = 65). Unstandardized coefficients with 95% confidence intervals are reported alongside standardized coefficients. Regression assumptions were examined: multicollinearity was acceptable (all VIF < 2.3), and residual normality was assessed by Shapiro–Wilk test and normal quantile plot, homoscedasticity by the Breusch–Pagan test and by inspection of residual-versus-fitted plots, independence by the Durbin-Watson statistic, and influential observations by Cook’s distance. Because the homoscedasticity assumption was violated and one observation proved highly influential (Section 4.2), three additional estimates of each coefficient are reported alongside the classical one: heteroscedasticity-consistent (HC3) standard errors, a percentile bootstrap over 5000 resamples of standardized coefficients, and a refit omitting the most influential case. We report the agreement and disagreement among these estimates rather than selecting one, because the stability of a coefficient across estimators is itself the relevant evidence at this sample size. To test the model-consistent specification directly, posttest UB was additionally regressed on FC.

Because the sample size was fixed by cohort enrollment, we conducted a sensitivity power analysis rather than a post hoc power calculation. With *N* = 65, four predictors, and α = 0.05, the design affords 80% power to detect an overall model effect of *f*^2^ = 0.20 and an individual predictor effect of *f*^2^ = 0.13 (approximately β = 0.30 at the observed level of collinearity). The two significant coefficients reported below exceed this threshold; the two nonsignificant coefficients fall below it, and should therefore be read as evidence that no effect of at least medium size was detected, not as evidence of absence. With *n* = 33, the paired comparisons afford 80% power to detect *d* = 0.50; the largest observed change (*d* = 0.36) falls below this value, and RQ1 is correspondingly underpowered for detecting small changes. Quantitative analyses used Python 3.13 (pandas 3.0, SciPy 1.18, statsmodels 0.15).

For RQ3, interview records were analyzed thematically (Braun & Clarke, 2006). Records were read repeatedly and coded inductively with sensitivity to the UTAUT factors, and codes were consolidated into themes through constant comparison across the two interview cycles. Initial coding was conducted by the first author with generative-AI assistance in identifying candidate theme occurrences across the records, and all coding decisions and theme definitions were made and verified by the first author. To support the trustworthiness of the analysis, an educational technology researcher with expertise in teacher education, who was not an author and was not involved in data collection, reviewed the codebook, representative coded excerpts, and the emerging theme structure; points of disagreement were resolved through discussion and the theme structure was revised accordingly. Because a single coder produced the initial codes, no inter-coder agreement statistic can be reported, and peer debriefing substitutes for, rather than equals, independent double coding (Section 6). Analysis proceeded across both cycles until no new themes were generated by additional records; we describe this as the point at which the theme structure stabilized across the corpus rather than claiming thematic saturation, since saturation was not formally assessed against a predetermined criterion. For each theme reported in Section 4.3, we state the number of interviewees whose accounts contributed to it and their distribution across the two cycles.

## 4. Results

### 4.1. Acceptance Before and After Use (RQ1)

Table 1 presents descriptive statistics and reliabilities for the full samples at each time point; Table 2 presents the analysis of change, which is based solely on the 33 students who responded at both time points. The two sets of figures answer different questions and should not be read as a single series.

In the full pre-use sample (*N* = 42), composite means ranged from 4.22 (EE) to 4.63 (BI): students entered the semester with strongly favorable expectations of the AI evaluation system. In the full posttest sample (*N* = 65), means ranged from 4.32 (EE) to 4.69 (BI).

In the matched sample, no factor declined and all six point estimates rose, but none of the six comparisons survived correction for multiple testing. Performance expectancy showed the largest change (*M* = 4.51 to 4.65; *t*(32) = 2.07, *p* = 0.047, 95% CI [0.00, 0.28], *d* = 0.36; Wilcoxon *p* = 0.055; Holm-adjusted *p* = 0.282). Changes in the remaining factors were small (all *d* ≤ 0.27) and nonsignificant by every criterion (Table 2). Because the confidence interval for the performance-expectancy difference includes values close to zero, because the parametric and nonparametric tests disagree at the conventional threshold, and because the Holm-adjusted value is far from significance, this increase should be treated as suggestive rather than as an established finding. The sensitivity analysis using the stricter pre-use cutoff produced a larger estimate (*n* = 28; *d* = 0.50, *p* = 0.014, Wilcoxon *p* = 0.024), consistent in direction but based on a smaller sample.

The more robust result concerns the absence of decline. Post hoc, exploratory one-sided non-inferiority tests, whose equivalence bound was not prespecified, rejected a decline of *d* = −0.35 or larger for every one of the six factors (all *p* ≤ 0.007; PE *p* < 0.001), and the upper bound of every confidence interval was positive. Acceptance was therefore stable across the semester in a sense stronger than the mere failure to reject a null hypothesis of no change: the data are inconsistent with any appreciable decline. This is a statement about the stability of acceptance scores. Whether that stability reflects the confirmation of prior expectations is a separate question, which the quantitative data cannot answer and which we take up in Section 5.1 using the interview evidence.

Effort expectancy was the lowest-rated factor at both time points (pretest *M* = 4.22, posttest *M* = 4.32 in the full samples). At the item level, the lowest posttest items concerned whether the usage process was uncomplicated (*M* = 4.25), whether the tool could be used without technical support (*M* = 4.25), and whether the interface was easy to navigate (*M* = 4.28), indicating that within a uniformly high profile the relatively weakest element of acceptance concerned usability rather than usefulness.

### 4.2. Factors Associated with Behavioral Intention After Sustained Use (RQ2)

Table 3 presents posttest inter-factor correlations, all positive and substantial (*r* = 0.55–0.70). Table 4 presents the regression of posttest BI on the four UTAUT determinants (*N* = 65). The model accounted for a large share of intention variance (*R*^2^ = 0.62, adjusted *R*^2^ = 0.59, *F*(4, 60) = 24.17, *p* < 0.001). Under classical standard errors, effort expectancy (β = 0.37, *p* = 0.001) and social influence (β = 0.39, *p* = 0.002) were significantly associated with intention, whereas performance expectancy (β = 0.16, *p* = 0.169) and facilitating conditions (β = −0.01, *p* = 0.935) were not.

Regression diagnostics, however, qualify this result substantially, and we report them in full. Multicollinearity was acceptable (VIF 1.78–2.21) and residuals were approximately normal (Shapiro–Wilk *W* = 0.970, *p* = 0.112) and independent (Durbin-Watson = 1.95). Two assumptions were violated. Residual variance was not constant (Breusch–Pagan *LM* = 32.87, *p* < 0.001), which is expected given the ceiling-bounded distribution of the outcome, and one observation was highly influential (Cook’s *D* = 0.79, with a second at 0.77; both belong to the small number of respondents who rated the system below the scale midpoint on intention).

Table 4 therefore reports four estimates of each coefficient: classical OLS, heteroscedasticity-consistent (HC3) standard errors, a percentile bootstrap over 5000 resamples, and a refit excluding the single most influential case. The pattern across these estimates is the substantive result. Effort expectancy showed the most consistent evidence of association with intention across specifications: significant under classical standard errors, with a bootstrap confidence interval excluding zero, marginal under HC3 (*p* = 0.054), and significant when the influential case is removed (β = 0.30, *p* = 0.008). The social influence coefficient is not stable: it is significant under classical standard errors but not under HC3 (*p* = 0.183), its bootstrap interval includes zero, and it falls below significance when the influential case is removed (*p* = 0.078). Performance expectancy shows the reverse instability, reaching significance only when the influential case is excluded (β = 0.31, *p* = 0.020). Facilitating conditions are nonsignificant throughout.

We therefore draw a narrower conclusion than the classical estimates alone would license. Effort expectancy showed the most consistent evidence of association with continued-use intention across specifications in these data. The association for social influence is not robust to the estimator or to a single respondent, and should be treated as provisional pending replication in a larger sample. Because these data are cross-sectional and all variables were measured at the same time point, even the effort expectancy result describes an association and does not establish that ease of use produced the intention.

Two further observations follow. First, the model-consistent specification was tested directly: facilitating conditions were substantially related to reported use behavior (*r* = 0.65), and in the regression of UB on FC the coefficient was large and clearly significant (*B* = 0.61, 95% CI [0.43, 0.79], β = 0.65, *t*(63) = 6.79, *p* < 0.001, *R*^2^ = 0.42). This is consistent with the original UTAUT placement of FC as a determinant of use rather than of intention (Venkatesh et al., 2003). The null FC-BI coefficient therefore does not indicate that facilitating conditions are irrelevant to this system; it indicates that their association runs to use rather than to intention in these data.

Second, the nonsignificant coefficients should not be read as null effects. As reported in Section 3.7, the design could detect individual predictor effects of approximately β = 0.30 with 80% power; the performance-expectancy coefficient (β = 0.16) lies below that threshold and its confidence interval spans both negligible and moderate positive values. Effort expectancy, uniquely, exceeds that threshold in every specification.

### 4.3. Participants’ Accounts of Acceptance and Its Limits (RQ3)

Thematic analysis of the 20 interviews yielded five themes. The findings reported here are participants’ perceptions of the system and of their own learning; they are not measures of the system’s accuracy or of changes in teaching competence. For each theme we indicate the number of interviewees whose accounts contributed to it and their distribution across the two cycles. Cycle 1 interviewees were drawn in equal numbers from the two courses; Cycle 2 interviewees were ten students who had not been interviewed in Cycle 1.

*Evidence-grounded specificity was described as making the AI evaluation persuasive* (all 20 interviewees; C1 10, C2 10). Across both cycles, interviewees attributed their trust in the system to the specificity of its grounds, that is, rubric scores anchored in time-stamped moments and quoted utterances extracted from their own demonstrations. One student said the AI presented concrete reasons and time-stamped evidence from the video, which led her to accept and act on judgments she had initially found hard to accept (C1-S1); another said that feedback which clearly exposed the weak parts of her lesson felt comparable to a professor’s advice (C1-S2); and a third emphasized that the AI’s detailed explanation of why it gave a 4 rather than a 5 clarified the criteria themselves (C1-S3).

*Score discrepancies were described as prompting questioning, reflection, and conceptual change* (all 20; C1 10, C2 10). Interviewees described comparing their own scores with the AI’s as the moment the system became a thinking tool rather than a scoring machine. When the AI assigned lower scores than expected, students questioned it through the dialogue function, and in working through the discrepancy they re-examined their own assumptions. One student reported realizing through dialogue that assistive technology includes not only AAC but also wheelchairs and customized student desks, an expansion of a core professional concept (C1-S8); the same student also discovered that prompts she had considered sufficient were not systematically structured and redesigned her prompt hierarchy. Repeated use was associated with internalization of standards: one interviewee reported that using the system repeatedly led her to grasp what kind of evidence the AI demands (C2-S5). These are participants’ self-reports of conceptual change; the study did not assess conceptual change independently.

*Feedback was reported as carrying over into lesson preparation and re-teaching* (all 20; C1 10, C2 10). Interviewees described moving from preparation centered on lesson structure and sequence toward planning aligned with the evaluation criteria themselves (C2-S10), incorporating AI-identified weaknesses into improvement plans, and perceiving gains in their second demonstrations (C2-S4). The system’s consistency made this cycle visible: one student who repeated the same mistake in the second demonstration said that the AI’s flagging of the identical point again confirmed for her that the earlier feedback had been principled rather than arbitrary (C2-S1). Improvement across demonstrations was not measured; these are perceived gains.

*The system was described as complementing scarce instructor feedback* (18 of 20; C1 8, C2 10). Interviewees framed the system’s value against the scarcity of instructor attention. AI feedback was reported to be always available, one-to-one, and free of social exposure, with one student noting that she could do it comfortably on her own (C2-S6), complementing instructor feedback that had been intermittent or centered on lesson plans (C2-S3, C2-S4). At the same time, most interviewees did not regard the AI as a substitute: the value of instructor feedback that engages deeply with learner-specific context was still recognized (C1-S3), and several students proposed an explicit combination of the two (C1-S7).

*Context errors, inconsistency, and interface friction were described as the limits of acceptance* (18 of 20; C1 9, C2 9). The limits interviewees identified were intermittent failures to recognize the demonstration context, such as comments unrelated to the lesson topic (C1-S10); inconsistency of judgments across sessions, with one student reporting that scores kept changing even after she had followed the system’s earlier advice (C2-S8); dialogue responses that felt canned; and the initial complexity of the multi-step procedure, which one student described as feeling complicated at first but becoming familiar with use (C2-S1). The most-requested improvement in Cycle 1, having the AI analyze the lesson plan together with the video so that it understands the lesson’s intent, was implemented before Cycle 2, and Cycle 2 interviewees identified it as a driver of improved evaluation accuracy (C2-S9).

### 4.4. Integration of Quantitative and Qualitative Findings

Table 5 sets the two strands against one another for each research question. The strands converge on the primacy of effort expectancy: the survey located the relatively weakest scores and one of the two strongest associations with intention in the same factor, and the interviews located the limits of acceptance in procedural complexity, context errors, and cross-session inconsistency rather than in doubt about the system’s usefulness. They also converge on the stability of acceptance, though they establish different things about it: the survey establishes that scores did not decline, while the interviews supply the participants’ own account of why their favorable view was maintained. The strands diverge in one respect. The survey shows no reliable change in social influence, whereas interviewees rarely raised peer or instructor endorsement spontaneously; the strong regression association for SI thus rests on survey data alone and finds little corroboration in participants’ narratives, a divergence we return to in Section 5.2.

A sixth pattern cuts across the five themes and qualifies them. In seven of the 20 interviews (C1 4, C2 3), students described resolving a discrepancy by adopting the AI’s score rather than defending their own. The accounts are explicit: one student reported that she had never lowered a score, only kept it or raised it by one or two points to match the system (C1); another described being persuaded by an explanation she found more convincing than her own reasoning and setting her score to the AI’s (C1). These are the same students who elsewhere describe questioning the system, so deference and interrogation coexisted within individual accounts rather than dividing the sample. We treat this as the boundary of the collaborative review the system was designed to elicit, and return to it in Section 5.2 and Section 6.

## 5. Discussion

### 5.1. Stability of Acceptance Across a Semester of Consequential Use

The first contribution of this study is temporal. Participants held very high expectations of the AI-based microteaching evaluation and feedback system before using it, with factor means of 4.2 to 4.6 on a 5-point scale prior to any meaningful contact with the tool. Under expectation-confirmation theory, such conditions carry a high risk that actual experience will fall short of expectations, producing disconfirmation and reduced continuance intention (Bhattacherjee, 2001; Oliver, 1980). The scores do not show that pattern. Across a semester in which the system evaluated graded work, no acceptance factor declined, every point estimate rose, and post hoc, exploratory non-inferiority tests against a bound of *d* = −0.35 were rejected for all six factors (all *p* ≤ 0.007). The quantitative finding, stated precisely, is the stability of acceptance: whatever the semester did, it did not erode students’ favorable evaluation of the system.

Whether that stability constitutes the confirmation of prior expectations is an interpretive question, and the interviews are the evidence that bears on it. In participants’ accounts, what sustained their favorable view was not that the system was faultless; interviewees cataloged its context errors and inconsistencies without hesitation. What they described as sustaining it was that the system’s judgments were accountable. Scores arrived with time-stamped moments and quoted utterances from one’s own demonstration, and when a judgment was contested, the dialogue function produced reasons that could be examined, accepted, or rebutted. In these accounts, a favorable view was maintained through repeated encounters with justified individual judgments rather than granted to the system as a whole. This is an interpretation of participants’ self-reports, not a measured mechanism; confirmation, satisfaction, and perceived disconfirmation were not assessed as variables (Section 2.4).

Participants also described their acceptance as behaviorally anchored. They reported planning subsequent demonstrations around the criteria the system had made visible, and reported that when improvement, or a repeated error, registered in the next evaluation, the feedback loop itself became evidence that the system’s judgments were principled. If these accounts are taken at face value, they suggest a refinement worth testing in future work on expectation confirmation with evidence-generating AI tools: expectations inflated by public discourse may survive contact with an imperfect system when the system continuously displays the grounds of its judgments, allowing users to affirm the tool’s value locally despite peripheral disconfirmations. We offer this as a hypothesis generated by the qualitative strand, not as a finding established here. For teacher educators considering whether students’ initial enthusiasm for AI tools provides a durable basis for curricular investment, this pattern is moderately encouraging.

The near-ceiling baseline demands interpretive restraint in both directions. The modest size of the observed increases cannot be read as evidence that the system made little impression, because there was very little room on the scale for an increase to register. Conversely, sustained high scores partly reflect a cohort favorably disposed toward AI before any contact with this system, and prior experience with AI tools was not measured, so we cannot characterize that disposition further. It remains notable that, with baseline scores this high, the scale allowed considerably more room for decline than for increase, and no factor declined.

### 5.2. Ease of Use as the Factor Most Closely Associated with Intention

The regression results have implications for where design effort should be directed. In many educational technology contexts, performance expectancy is the dominant correlate of intention (Scherer et al., 2019; Venkatesh et al., 2003). That was not the case here, and the most plausible reason is statistical rather than substantive: virtually every respondent rated the system useful, so restriction of range left perceived usefulness unable to differentiate among students’ intentions. What did differentiate them was the lowest-rated factor, effort expectancy, with item-level weaknesses concentrated in an uncomplicated usage process, unsupported use, and interface navigation. Effort expectancy was also the predictor showing the most consistent evidence of association with intention across the estimators we applied, which is why we treat it, and not social influence, as the finding on which design recommendations may rest (Section 4.2). We note again that this is an associational finding from cross-sectional data.

This pattern is intelligible in light of the system’s three-stage workflow, in which the output of one stage must carry over into the next, and it recurs across all three of the data sources we collected. In the pre-implementation expert usability evaluation, the lowest-rated heuristic items, against an overall mean of 4.5, concerned precisely this region: predictive guidance toward the next step (*M* = 3.4), undo and redo control (*M* = 3.8), and error recognition, description, and recovery (*M* = 3.4–3.8) (G. Lee et al., 2025). Students surveyed in this study rated the same domain lowest, and students interviewed located the limits of acceptance in the same regions of residual friction: initial procedural complexity, intermittent context-recognition errors, and cross-session feedback inconsistency (Section 4.3). Several expert-recommended improvements were implemented before field use (G. Lee et al., 2025), and the lesson-plan analysis function added between cycles was credited by Cycle 2 interviewees with improved evaluation accuracy; yet residual friction persisted. Usability proved to be an iterative agenda rather than a one-time fix.

These three sources are distinct in method and in participant group—heuristic review by five external experts, a survey of student users, and interviews with student users—but they are not independent in the strict sense. The same research team designed the system, selected the heuristic instrument, wrote the survey items, and conducted the interviews, and the two student-facing sources draw on overlapping participants. Their convergence therefore strengthens confidence that the identified weak spots are real, but it does not constitute triangulation by mutually independent observers. With that qualification, the design agenda is clear: streamline stage transitions and file handling, provide anticipatory guidance about the next action, and build self-service support that removes dependence on technical assistance. The system has established, in its users’ eyes, that it is worth using; it has not yet established that it is easy to use, and it is perceived ease of use that is most closely associated with the intention to continue.

The regression association for social influence requires a more guarded reading than it might otherwise receive. The first reason is statistical: as reported in Section 4.2, the coefficient is significant under classical standard errors but not under heteroscedasticity-consistent estimation, its bootstrap interval includes zero, and it loses significance when a single influential respondent is removed. Three further reasons are specific to this setting. Use of the system was a requirement of two courses, so the normative environment was not a variable that students encountered with any latitude; institutional endorsement was unambiguous for everyone in the sample. The courses were taught by instructors associated with the system, which plausibly raised the perceived endorsement of important others while simultaneously creating conditions in which students might report favorable views for reasons unrelated to the system’s merits. And the authors developed the system, a conflict disclosed at the end of this article. It is also notable that interviewees rarely raised peer or instructor endorsement spontaneously (Section 4.4), so the SI finding rests on a survey estimate that is itself unstable. The observation that the social environment matters is consistent with UTAUT and with the broader literature (Scherer et al., 2019; Venkatesh et al., 2003), and the practical implication—that instructors who visibly engage with AI feedback, discuss it in class, and model its integration with professional judgment help construct the normative environment on which continued use depends—remains reasonable. But the magnitude of the SI coefficient in this study should not be generalized to voluntary-use settings.

Students’ accounts also give empirical content to the human–AI collaboration competencies around which the system was designed. Questioning a score, demanding its evidentiary grounds, checking the flagged scene, and then accepting, adjusting, or rejecting the judgment correspond to the collaborative task regulation and calibrated, rather than blind, trust that the collaboration literature identifies as core competencies for working with AI (Y. J. Yoo et al., 2024), and that the system’s dialogue-based review structure was intended to elicit (G. Lee et al., 2025). At the same time, seven of the 20 interviewees described deferring to the AI when its scores diverged from their own, typically by revising their own score upward to match it (Section 4.4), which marks the boundary of this enactment. Prior work on this cohort supplies a quantitative counterpart to these accounts. In the design and development strand conducted with a subset of these students, self-assessments and instructor assessments of the same demonstrations converged closely in the second cycle: composite means of 4.26 and 4.30, respectively, a mean absolute difference of 0.76 on the rubric scale, and a signed bias of −0.04, with 22 of 33 students classified as accurate, six as underestimating, and five as overestimating (Y. Yoo, 2026). Two interpretations should be distinguished. Convergence of this degree is consistent with students having internalized the criteria, which is what the interviewees in the present study describe. It is also consistent with students having adopted the system’s frame of reference, which is what deference would look like from the outside. The present data cannot distinguish them, and the near-absence of systematic bias in either direction does not settle the question, because a student who defers and a student who has calibrated will both agree with the evaluation.

Critical trust, these cases suggest, may not emerge automatically from a well-designed interface; programs adopting AI-supported evaluation might therefore treat evidence-questioning routines as instructable components of professional judgment rather than as optional system features. Whether such instruction improves calibration is an open question this study cannot answer.

### 5.3. Implications for Special Education Teacher Preparation

The implications drawn here concern the acceptance of AI-supported evaluation in special education teacher preparation. This study did not assess the validity of the system’s evaluations, changes in participants’ teaching competence, or the pedagogical effectiveness of the system, and no claim about those matters follows from our data.

The special education context sharpens the findings in two ways. First, special education’s evaluation criteria are broader and more differentiated in ways that tax human raters (Bejar, 2012; S. H. Lee et al., 2024), which means that a tool offering consistent application of such criteria addresses a salient need; participants’ sustained performance expectancy is consistent with their recognition of that need, although we did not test whether the system in fact delivers consistency. Field demand for AI tools attuned to special education contexts is growing (Salas-Pilco et al., 2022). Second, interviewees’ accounts suggest that the rubric’s special education alignment component (G. Lee et al., 2025) was noticed and engaged with; it was in dialogue over a contested score that one student reported her conception of assistive technology expanding beyond AAC to include positioning equipment and customized desks (Section 4.3). This is a single self-reported instance, and it indicates that special education-specific criteria embedded in an AI evaluation can become an object of student reflection; it does not establish that such criteria produce conceptual change, which would require a design capable of measuring that change.

At the same time, special education programs emphasize accessibility as a professional value. A tool perceived as workable only with technical support sits uneasily in a curriculum that teaches universal design. Improving the system’s effort expectancy is therefore not merely an adoption strategy but a matter of pedagogical coherence: an evaluation tool should itself embody the accessibility principles the program teaches.

### 5.4. Implications for Acceptance Research

Methodologically, this study shows what single-time-point acceptance surveys conceal. Had only the posttest been administered, the high means would plausibly have been attributed to the semester’s experience. The pretest reveals that acceptance was largely imported into the course, and that the semester’s contribution was the maintenance of an already favorable view rather than its creation.

In cohorts saturated with AI discourse, acceptance researchers should anticipate compressed, ceiling-adjacent distributions and design accordingly: preferring pre–post and longitudinal designs, item formats with more headroom, and behavioral usage indicators alongside self-reports. Our experience also suggests two specific procedural recommendations: measuring confirmation and satisfaction directly if expectation-confirmation claims are to be made quantitatively, and reporting equivalence or non-inferiority tests rather than treating a nonsignificant difference as evidence of stability.

## 6. Limitations and Future Research

Several limitations qualify these conclusions. We group them by source and pair each with the design feature that would address them.

Design and inference. The single-group design without a comparison group cannot rule out maturation or course-related influences on posttest responses. A nonsignificant difference is not, by itself, evidence of stability. The non-inferiority tests reported in Section 4.1 address this directly by testing against a specified bound rather than against a null of exact equality, but their bound was not prespecified, so they are post hoc and exploratory; they establish only the absence of an appreciable decline within this single group, and maturation could in principle offset a decline the system would otherwise have produced. Future work should incorporate a comparison group or a staggered rollout, in which cohorts begin using the system at different points, so that change can be attributed with greater confidence.

Statistical assumptions. The regression residuals were heteroscedastic (Breusch–Pagan *p* < 0.001), a consequence of the ceiling-bounded outcome distribution, and two observations exerted substantial influence (Cook’s *D* = 0.79 and 0.77). Under heteroscedasticity-consistent standard errors no predictor reached conventional significance, and the identity of the significant predictors changed when influential cases were removed. We therefore report the full set of estimates in Table 4 rather than a single preferred model, and we limit our substantive claim to effort expectancy, the coefficient showing the most consistent evidence of association across specifications. A larger sample, and a response format with more headroom to reduce ceiling-induced heteroscedasticity, are the corresponding remedies.

Measurement. We used an adapted UTAUT instrument whose factor structure was not validated in this sample; no confirmatory factor analysis was feasible at *N* = 65 (Section 3.4). Pre–post comparability is also imperfect: items were phrased prospectively at pretest and retrospectively at posttest, measurement invariance could not be tested, and the use behavior factor compared anticipated with enacted use. All data are self-reported, and use behavior was measured by self-report items rather than system logs. Future administrations should validate the adapted instrument in a larger sample, test longitudinal measurement invariance, and triangulate self-report with system log data and with evaluation-quality indicators such as AI–expert rating agreement.

Sample and power. The sample was drawn from a single program at a single Korean university, and the matched subsample (*n* = 33) is small and may overrepresent students engaged enough to complete both time points. As reported in Section 3.7, RQ1 was underpowered for changes smaller than *d* = 0.50, and RQ2 could not detect predictor effects below approximately β = 0.30; the nonsignificant coefficients for performance expectancy and facilitating conditions should be read accordingly. Ceiling effects further compress variance and attenuate change estimates. Future work should recruit across multiple programs and institutions, and should use response formats with greater headroom—finer-grained scales or forced-choice comparative formats—to reduce ceiling compression.

Procedural and ethical issues. The pretest survey was not closed before system use began, and 37 responses submitted after use onset had to be excluded (Section 3.2); under the rotating group schedule, some retained responses may also sit close to individual students’ first system contact. A sensitivity analysis with a stricter pre-use cutoff yielded the same conclusions with a larger performance-expectancy effect. More seriously, the pre-use survey was administered before the date of final ethics approval and outside the data-collection window specified in the approved protocol. The research team is aware of this; consent was obtained at the outset of data collection and again, on the approved form, after final approval (Section 3.2), but the findings based on the pre-use measure should be read with that qualification. Future administrations should complete and close pre-use measures before students’ first use of the system and align data-collection windows with the approval date.

Implementation heterogeneity. The system was used in two courses that differed in instructor, subject matter, and enrolled students, and the version used in Cycle 2 differed from that used in Cycle 1 (Section 3.6). Posttest acceptance therefore refers to the system as experienced across a changing implementation, and the course could not be tested as a moderator at this sample size. Future work should either hold the system version constant across the observation period or treat version as an explicit factor, and should recruit samples large enough to test instructor and course effects.

Compulsory use and researcher involvement. Use was a course requirement, so reported intention cannot be read as free choice, and social influence was measured where institutional endorsement was unambiguous (Section 5.2). The course instructors were associated with the system and the authors developed it, so social desirability may have inflated scores. Future work should examine acceptance in voluntary-use settings and in implementations run by instructors with no connection to the development team, and should collect acceptance data through channels administered independently of the course.

Qualitative strand. The interview records combine theme-level summaries with partial verbatim transcription rather than full transcripts, which limits the traceability of interpretations to the data. Initial coding was conducted by a single author; peer debriefing by an external researcher supports trustworthiness but is not equivalent to independent double coding, and no inter-coder agreement statistic can be reported. We describe the theme structure as having stabilized across the corpus rather than claiming thematic saturation, which was not assessed against a predetermined criterion. Interviews were conducted by a member of the development team, which may have moderated criticism. Interviewees were volunteers rather than a purposively selected sample, so the accounts in Section 4.3 are subject to self-selection: students who declined to be interviewed may have held views the themes do not represent. Future work should transcribe in full, employ at least two independent coders with a reported agreement statistic, use an explicit saturation criterion, engage an interviewer external to the development team, and sample interviewees purposively across the range of survey responses rather than relying on volunteers.

Overreliance. Seven of the 20 interviewees described deferring to the AI when its scores differed from their own, and the direction was consistent: they raised their own scores to meet the system’s rather than lowering the system’s to meet theirs. This should be examined as a potential overreliance risk rather than read solely as evidence of acceptance, and future work should measure agreement between student and AI judgments directly and test whether instruction in evidence-questioning routines improves calibration.

Beyond addressing these limitations, future research should extend the observation period beyond a single semester, since distinguishing maintained acceptance from an extended novelty effect requires longer time series; deepen qualitative inquiry into how students enact AI feedback in subsequent planning; and test whether the usability-dominant association pattern replicates in cohorts with more heterogeneous prior attitudes toward AI.

## 7. Conclusions

Preservice special education teachers began the semester with strongly favorable expectations of an AI system that evaluates and gives feedback on their teaching demonstrations, and those expectations were maintained rather than eroded across a semester of sustained use in which the demonstrations evaluated were themselves graded coursework, although the system’s output did not contribute to grades. Post hoc, exploratory non-inferiority tests indicate that acceptance did not decline appreciably on any factor, a reading that awaits confirmation in a study with a prespecified margin. Participants’ own accounts, which attribute this stability to the system’s practice of displaying the grounds of its judgments, offer an interpretation of that stability in expectation-confirmation terms; confirmation itself was not measured, and this interpretation rests on the interview data rather than on the survey.

After sustained use, perceived ease of use showed the most consistent evidence of association with the intention to continue using the system. Perceived usefulness was uniformly high and therefore did not differentiate among students, and the apparent association with perceived social support did not survive robust estimation or the removal of a single influential respondent, so we report it as provisional. Because the regression is cross-sectional, these are associations rather than demonstrated causes.

These findings come from a single program, with *N* = 65 at posttest and *n* = 33 in the matched-pair analysis, in a setting where use was compulsory and the instructors were associated with the system; they should be treated as a first field account rather than as a basis for general recommendations. Within those bounds, they suggest a division of responsibility that merits examination in other settings: improving usability is a task for developers, while establishing a normative environment in which AI-supported evaluation is modeled, discussed, and integrated with professional judgment is a task for teacher educators. As AI systems take on evaluative roles in teacher preparation, the question for programs may be shifting from whether preservice teachers will accept them to how programs will make their sustained use easy and professionally legitimate.

## Figures and Tables

**Table 1:** Factor composition and internal consistency.

Factor	Items	α (Pre)	α (Post)
Performance expectancy (PE)	6	0.90	0.90
Effort expectancy (EE)	5	0.89	0.90
Behavioral intention (BI)	4	0.95	0.92
Social influence (SI)	3	0.84	0.87
Facilitating conditions (FC)	3	0.82	0.88
Use behavior (UB)	2	0.86	0.82

*Note.* Pretest *N* = 42, posttest *N* = 65 (full samples); 5-point Likert scale. No factor analysis was conducted; see Section 3.4.

**Table 2:** Pre–post comparison of UTAUT factors (paired sample, *n* = 33). Descriptive statistics for the full samples are given in the text and differ from the matched-sample values below.

Factor	Pre *M (SD)*	Post *M (SD)*	*t*(32)	*p*	Holm *p*	Wilcoxon *p*	*d* [95% CI of MD]	Non-Inf. *p*
PE	4.51 (0.52)	4.65 (0.46)	2.07	0.047	0.282	0.055	0.36 [0.00, 0.28]	<0.001
EE	4.19 (0.83)	4.27 (0.62)	0.61	0.546	1.000	0.586	0.11 [−0.17, 0.32]	0.007
BI	4.64 (0.65)	4.76 (0.48)	1.56	0.130	0.648	0.091	0.27 [−0.04, 0.26]	0.001
SI	4.49 (0.59)	4.54 (0.57)	0.63	0.530	1.000	0.512	0.11 [−0.11, 0.21]	0.006
FC	4.49 (0.61)	4.56 (0.55)	0.73	0.472	1.000	0.350	0.13 [−0.13, 0.27]	0.005
UB	4.53 (0.72)	4.67 (0.50)	1.27	0.213	0.850	0.193	0.22 [−0.08, 0.36]	0.001

*Note. d* is Cohen’s *d* for paired samples; positive values indicate higher posttest scores. MD = mean difference. Holm *p* = Holm–Bonferroni adjusted *p* across the six comparisons. Non-inf. *p* = one-sided test against the lower bound *d* = −0.35. UB compares anticipated use at pretest with self-reported use at posttest and is not interpreted substantively (Section 3.4).

**Table 3:** Posttest inter-factor correlations (*N* = 65).

	PE	EE	SI	FC	BI
EE	0.55	-			
SI	0.67	0.58	-		
FC	0.65	0.60	0.63	-	
BI	0.62	0.67	0.70	0.56	-
UB	0.65	0.65	0.65	0.65	0.69

*Note.* All correlations significant at *p* < 0.001.

**Table 4:** Regression on posttest behavioral intention (*N* = 65).

Predictor	*B* [95% CI]	β	*p* (OLS)	*p* (HC3)	Bootstrap 95% CI (β)	β (Excl. Case)
PE	0.20 [−0.09, 0.48]	0.16	0.169	0.413	[−0.15, 0.53]	0.31 *
EE	0.28 [0.12, 0.44]	0.37	0.001	0.054	[0.03, 0.61]	0.30 **
SI	0.36 [0.14, 0.58]	0.39	0.002	0.183	[−0.01, 0.89]	0.23
FC	−0.01 [−0.24, 0.22]	−0.01	0.935	0.954	[−0.30, 0.28]	0.08

*Note. R*^2^ = 0.62, adjusted *R*^2^ = 0.59, *F*(4, 60) = 24.17, *p* < 0.001. VIF 1.78–2.21. HC3 = heteroscedasticity-consistent standard errors. Bootstrap = 5000 percentile resamples of standardized coefficients. “excl. case” = refit omitting the single most influential observation (Cook’s *D* = 0.79; *N* = 64); * *p* < 0.05, ** *p* < 0.01. The FC-BI path is exploratory and departs from the original UTAUT specification (Section 2.4).

**Table 5:** Joint display of quantitative and qualitative findings by research question.

Research Question	Quantitative Finding	Qualitative Finding	Relation
RQ1: Level and change in acceptance	High at both time points; no factor declined; post hoc, exploratory non-inferiority against *d* = −0.35 rejected for all six factors; PE increase suggestive only	Participants described their favorable view as maintained through evidence-grounded scoring, dialogue over discrepancies, and visible uptake into re-teaching	Convergent; the qualitative strand supplies the interpretive account that the quantitative strand cannot
RQ2: Correlates of intention	EE and SI associated with BI; PE and FC not; FC associated with UB	Limits of acceptance located in procedural complexity, context errors, inconsistency; usefulness not questioned	Convergent on EE; divergent on SI, which interviewees rarely raised
RQ3: Accounts of acceptance	EE lowest-rated factor; item-level weaknesses in unsupported use and navigation	Initial procedural complexity, dependence on support, interface friction	Convergent at item level

## Data Availability

The data presented in this study are available on request from the corresponding author due to privacy and ethical restrictions.
